# Medicinal Plants Used to Manage Human and Livestock Ailments in Raya Kobo District of Amhara Regional State, Ethiopia

**DOI:** 10.1155/2020/1329170

**Published:** 2020-10-29

**Authors:** Ashenafi Osman, Desta Berhe Sbhatu, Mirutse Giday

**Affiliations:** ^1^College of Natural and Computational Sciences, Mekelle University, P.O. Box 231, Mekelle, Ethiopia; ^2^Mekelle Institute of Technology, Mekelle University, P.O. Box 1632, Mekelle, Ethiopia; ^3^Aklilu Lemma Institute of Pathobiology, Addis Ababa University, Addis Ababa, Ethiopia

## Abstract

Plant-based traditional medicine is practiced in Raya Kobo district, Amhara Regional State, Northeastern Ethiopia, to manage different human and livestock ailments. However, the formal ethnobotanical survey that documented such knowledge is lacking. Therefore, the aim of this study was to document the traditional knowledge on the use of medicinal plants to manage human and livestock ailments in the district. The study was conducted from January to July 2017 in five purposefully selected kebeles of the district. Ethnobotanical data were collected mainly using semistructured interviews conducted with 150 informants. In the five kebeles, 30 informants (15 males and 15 females) were selected using the stratified random sampling method from a list of traditional practitioners and knowledgeable individuals. Data were analyzed by employing descriptive and inferential statistical methods. The study documented a total of 91 medicinal plant species (distributed in 51 families) used in managing 38 human and 12 livestock ailments. Out of the total recorded plants species, 74 and 17 were used in managing human and livestock ailments, respectively. Leaves were the most frequently used plant parts in the preparation of remedies, accounting for 53.1% of the total preparations. The three most common methods of remedy preparation were grinding/pounding (23.5%), crushing (19.8%), and boiling (14.5%). Preference ranking conducted by selected informants on eight medicinal plants used in treating human febrile illness locally called “mich” revealed that *Ocimum urticifolium* is the most preferred medicinal plant—an indication of its high potency against the disease, and therefore needs to be prioritized for future scientific investigation. The result of this study demonstrated the rich traditional knowledge and practices in the district on the use of medicinal plants in treating various human and livestock ailments. Deforestation and drought were reported to be the major factors in the district threatening the medicinal plants and the associated knowledge. Thus, concerted efforts have to be made to conserve this important heritage using every possible means.

## 1. Background

Estimates show that 80% of the population living in developing countries depends on traditional medicine for its healthcare needs [1]—a practice that largely relies on the use of plants. The high prevalence in the use of traditional medicine is mainly attributed to its low cost, efficacy, and better accessibility. Traditional medicine is also serving as a source of knowledge in the development of many plant-based synthetic drugs—e.g., morphine used as analgesic is synthesized from *Papaver somniferum*, aspirin used as analgesic is synthesized from *Filipendula ulmaria*, and quinine used in treating malaria is synthesized from *Cinchona pubescens* [2].

In Ethiopia, the knowledge and utilization of traditional medicine, in general, and medicinal plants, in particular, are believed to be wide due to high diversity of higher plants, estimated to reach 6,000 species [3], and rich cultures, belief systems, and languages. One report indicated that about 80% of the Ethiopian population is still dependent on traditional medicine principally using plants [4]. Abebe and Ayehu [5] reported the application of more than 800 medicinal plant species in the Ethiopian traditional medicinal system. Traditional medicine is the most affordable and easily accessible source of treatment in the primary healthcare system of many communities of the country. But the rich knowledge of traditional medicine that has been developed over thousands of years is being exposed to serious depletion mainly due to deforestation, environmental degradation, overexploitation, agricultural land expansion, acculturation, and limited practice of its documentation.

In Ethiopia, cognizant of the role of traditional medicine and medicinal plants and the existing threats to the associated knowledge and practices, attempts have been made, mainly in the last four decades, to document medicinal plants used by many communities. Some of the notable works include that of Giday et al. [6] (in the northwestern part), Giday and Ameni [7] (in the northern part), Gedif and Hahn [8], and Teklehaymanot et al. [9] (in the central part), Wondimu et al. [10] (in the southeast part), and Abbink [11] (in the southwestern part). However, such works are not inclusive; thus, concerted efforts should be made in the country to document such valuable knowledge for better utilization and conservation.

A number of ethnobotanical studies have been conducted in different districts of the Amhara State of Ethiopia to which the Raya Kobo district belongs [6, 12–29]. There are 11 rural districts in the North Wollo zone of the Amhara State including Raya Kobo. However, the survey of published works shows that ethnobotanical studies on medicinal plants were conducted in only 2 of the 11 districts in the zone, namely, in Guba Lafto and Delanta. The studies in Guba Lafto [26] and Delanta [24] districts reported 135 and 133 medicinal plant species, respectively. People in the Raya Kobo district, like in other communities in Ethiopia, are expected to have been heavily dependent on herbal medicine to manage human and livestock ailments. However, an ethnobotanical study that aims to document the local use of medicinal plants is lacking. Thus, the aim of this study was to document the local knowledge on the use of medicinal plants in treating human and livestock ailments.

## 2. Materials and Methods

### 2.1. Description of the Study Area

**Figure 1:**
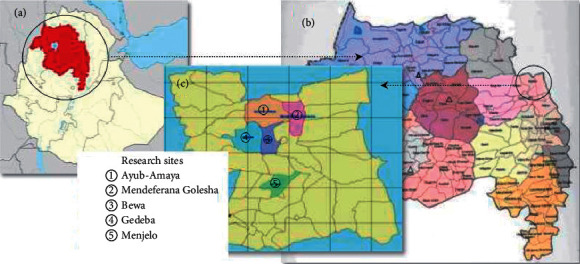
Research site: (a) Ethiopia, (b) Amhara State, and (c) Raya Kobo district (not legal map).

The study was carried out in Raya Kobo district of North Wollo zone of the Amhara State of Ethiopia (Figure 1). It is located between 11^○^54′ 04″ and 12^○^02′ 56″N latitudes and 39^○^25′ 56″ and 39^○^ 49′ 04″E longitudes. Kobo is the administrative town of the district and is located at 570 km north of Addis Ababa. Raya Kobo is bordered by Raya Alamata district of Tigrai State in the north, by Guba Lafto and Gidan districts of Amhara State in the south and west, respectively, and by Golina district of Afar State in the east. The district covers a total area of 183,697.50 hectares of land, of which 59% is kola (lowland), 38% is woyinadega (semihighland), and 3% is dega (highland) with annual temperature ranging from a minimum average of 12.31°C to a maximum average of 33.07°C and annual rainfall ranging from 500 mm to 800 mm [30]. The district is divided into 44 rural and four urban kebeles (subdistricts). Kebele is the smallest administrative unit in Ethiopia.

Raya Kobo district has a total population of 261,897 (females 128,157; males 133,740) [31]. Amharic is the mother tongue language of the great majority of the people of the district [32]. According to a recent local government data, 28% of the district is barren land, 23.7% is agricultural/cultivated land, 24.3% is grazing land, and 15.9% is covered by shrubs and bushes. Agriculture (crop and livestock production) is the livelihood activity of the great majority of people. Crops including sorghum, teff, and pulses are the most commonly cultivated ones. Likewise, it has high populations of sheep (201,753), chickens (183,261), and cattle (158,370). Furthermore, it is home to 47,951 goats, 22,232 donkeys, 13,864 camels, 552 mules, and 48 horses [33].

It was reported that there are seven health centers and 43 health posts in Raya Kobo. Malaria, common cold, tuberculosis, mich (febrile illness), pneumonia, HIV-AIDS, nekersa (chronic wound), hepatitis, diarrhea, typhoid fever-typhus, and diabetics are the top ten diseases of major public health priorities in the district [34]. Unpublished 2019 government data revealed that blackleg, anthrax, pasteurellosis, sheep and goat pox, lumpy skin disease, mange, tick infestation, and internal parasitic infections are the top eight diseases of veterinary health priorities. But, only 24 veterinary clinics were operating in 2019 [33].

### 2.2. Selection of Study Kebeles and Informants

Five kebeles, namely, Menjelo, Bewa, Gedeba, Ayub-Amaya, and Mendeferana Golesha (Figure 1), were purposively selected for the study following the approach of Martin [35] with the assistance of district authorities, elders, and knowledgeable persons based on the availability of traditional practitioners and knowledgeable individuals. For this purpose, many individuals, aged 40 years and above who claimed to be practitioners of traditional medicine and believed to be knowledgeable, were identified from each of the five kebeles. Then, 30 informants (15 males and 15 females) were selected from each kebele using a stratified random sampling approach [36].

### 2.3. Collection of Ethnobotanical Data

Ethnobotanical data were collected between January and July 2017 mainly through (a) semistructured interviews conducted with the sampled informants using a list of questions prepared beforehand in Amharic, mother tongue language in the study district, and (b) field observations using the procedures recommended by Martin [35] and Alexiades [37]. Gathered data and information were local names of medicinal plants, plant part(s) utilized, method of preparation, ailments treated or prevented, and routes of administration. Specimens of most of the medicinal plants were collected and identified by botanists at the Ethiopian National Herbarium (ETH) and Aklilu Lemma Institute of Pathobiology (ALIPB), Addis Ababa University, Ethiopia, and vouchers were deposited at the Endod and Other Medicinal Plants Research Unit, ALIPB.

### 2.4. Simple Preference Ranking

Preference ranking was performed by seven informants on eight medicinal plants used in treating mich (a human febrile illness) following the guideline established by Martin [35]. Informants for this exercise were randomly selected from those individuals already involved in semistructured interviews. The eight plants had the highest informant consensus among a total of 13 plants reported to be used in treating mich. Of all the ailments reported, mich was found to be treated by the highest number of medicinal plants—an indication of the high health importance of the ailment in the district. The highest value (i.e., 8) was given to medicinal plants considered to be the most effective, and the least value (i.e., 1) was given to the plant considered as the least effective.

### 2.5. Data Analysis

Descriptive statistical methods were used to produce frequencies and percentages. Coefficient of correlation and analysis of variance (ANOVA) were used to compare mean values of knowledge of medicinal plants among different groups of informants.

## 3. Results and Discussion

### 3.1. Diversity of Medicinal Plants

**Figure 2:**
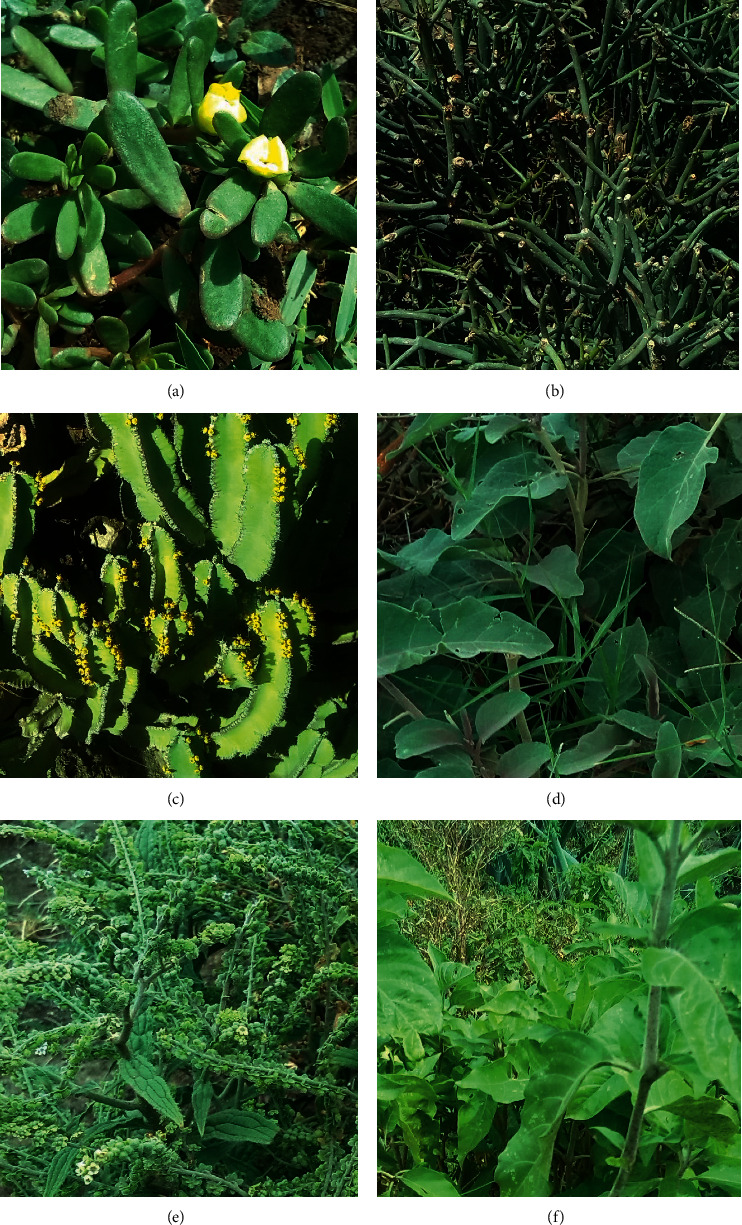
Some medicinal plants of Raya Kobo. (a) Antaria (*Portulaca oleracea*); (b) kinchibt (*Euphorbia tirucalli*); (c) qulqualda (*Euphorbia spp.*); (d) embuay (*Cucumis dipsaceus*); (e) yegid zemedie (*Cynoglossum lanceolatum*); and (f) simiza (*Justicia schimperiana*).

The present study documented a total of 91 species of medicinal plants. Whereas 74 of the species were used to manage human health problems only (Table 1), and 17 of them were used against livestock ailments only (Table 2). Some examples are given in Figure 2. As reflected by the richness of the plants used and the diversity of ailments treated, the results showed the profound roles of medicinal plants in meeting the basic healthcare needs of the communities in the study area. Previous studies carried out in Delanta and Guba Lafto districts of North Wollo zone, to which the study district belongs, reported the use of 135 and 133 species of medicinal plants, respectively, to manage various ailments [24, 26]. Of the reported medicinal plants, 42 (46.2%) were herbs, 29 (31.8%) were shrubs, and 20 (22%) were trees. Previous ethnobotanical studies conducted elsewhere in several districts of Ethiopia also revealed the wide use of herbs as medicinal plants [14, 38–40]. Such wide use of herbs in the study district may be attributed to their ease of harvesting and processing and their relatively better abundance compared to shrubs and trees as it was observed by the investigators and documented in the local government records [33].

The reported medicinal plants belonged to 51 families and 83 genera. Whereas six species belonged to Fabaceae, five species belonged to each of the families of Asteraceae, Euphorbiaceae, and Lamiaceae. Solanaceae was represented by four species, while Boraginaceae, Cucurbitaceae, Ranunculaceae, and Vitaceae were represented by three species each. Each of the remaining 42 families was represented by two or one species. The fact that Fabaceae, Asteraceae, Euphorbiaceae, and Lamiaceae contributed higher number of medicinal plants could probably be attributed to their better species richness in the flora of Ethiopia. Fabaceae, Asteraceae, Euphorbiaceae, and Lamiaceae are among the largest families in the flora of Ethiopia and Eritrea containing 486, 440, 209, and 184 species, respectively [41–44].

### 3.2. Ailments Managed by Medicinal Plants

The medicinal plants documented by the present study were used for treating 38 human ailments (Table 3) and 12 livestock diseases (Table 4). Of the 74 medicinal plants used for treating human ailments, 17, 13, and 10 were used to treat gastrointestinal complaints, mich, and wound, respectively; while snake bite and devil's illness were found to be treated by six medicinal plants each. Each of the remaining 33 human ailments was reported to be managed by four or fewer medicinal plants (Table 3). Diarrhea, mich, and wound were among the top ten diseases of major public health importance in the study district [34]. Mich is a local term used by traditional medicine practitioners for human ailment mainly characterized by fever, headache, and sore lips but not well-understood and recognized by modern healthcare practitioners. The fact that relatively high number of medicinal plants is used for treating mich in the district could be an indication of the high health importance of the ailment and the lack of effective treatment against it in modern medicine.

Of the 17 medicinal plants used for treating livestock diseases, three medicinal plants were used to treat dislocated/broken bones and three more to treat leech infestation. Likewise, mange, insect bites, and wound are treated by two medicinal plants each; while the remaining seven ailments are treated by one medicinal plant each (Table 4). According to an unpublished 2019 local government data, mange and anthrax were reported as the top two of the eight diseases of veterinary importance in the district [34].

### 3.3. Plant Parts Used and Methods of Remedy Preparation and Administration

**Figure 3:**
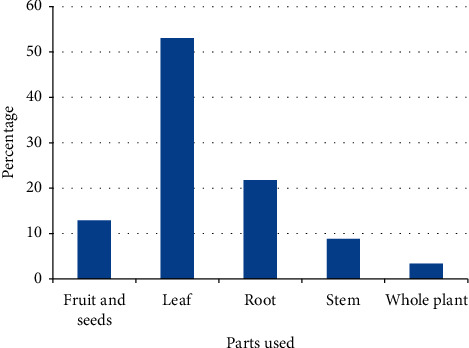
Proportions of plant parts used in preparation of remedies in Raya Kobo district.

Leaves were the most commonly used plant parts in the preparation of remedies in Raya Kobo accounting for 53.1% of the total preparations, followed by roots (21.8%) and fruits and seeds (12.9%) (Figure 3). Ethnobotanical studies carried out elsewhere in Ethiopia [20, 38, 40, 45] also reported that leaves are the most widely used parts in the preparation of plant remedies. The wide use of leaves in the preparations of plant remedies may be attributed to the fact that leaves are much easier to process quickly as compared to other plant parts. Collection of leaves does not pose a great danger to the survival of individual plants as compared to the collection of underground parts, stems, and whole plants. Removal of up to 50% of leaves of plants does not significantly affect their growth [36]. Contrary to ours, studies conducted in other places of Ethiopia [46, 47] observed roots as the most widely used plant parts in preparing traditional remedies. Harvesting roots, if not performed carefully, may cause detrimental effects on the plants that could ultimately bring about their disappearance or extinction from their natural habitats [6, 48].

The preparation methods of plant remedies in the study district were diverse. The most commonly used methods were grinding/pounding (23.7%), crushing (19.8%), boiling (14.5%), chewing (10.7%), squeezing (8.4%), and cutting and bleeding (6.9%) (Table 5). Similar studies conducted in the country by Girmay and Teshome [49], Amenu [40], and Hunde et al. [47] reported crushing, grinding, or pounding as the most common methods of preparation of plant remedies. But Lulekal et al. [46] reported boiling as the most frequently used method of remedy preparation in Mana Angetu district of Oromia State of Ethiopia. Choice of preparation methods may be influenced by the types and diversity of medicinal plants as well as the cultural background of the communities practicing traditional medicine.

**Figure 4:**
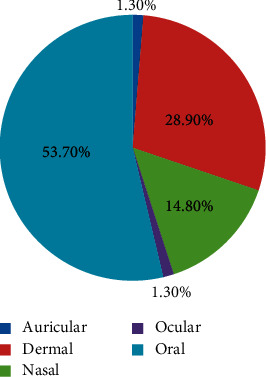
Frequency distribution of administration route of remedies in Raya Kobo district.

Analysis of the routes of administration revealed that the majority of the plant remedies were given orally (53.7%), followed by those applied dermally (or topically) (28.9%) (Figure 4). Similar observations were reported by many studies carried out in other parts of Ethiopia [14, 38, 46, 49–53]. Ashagre [54] reported that both oral and dermal routes permit the quick physiological reaction of remedies to the causative agents of diseases to increase curative power. In the present study, informants affirmed that they advise their patients to be cautious during and after application of remedies depending on the types of ailments treated. For instance, in some cases, patients were restricted from drinking milk and local alcoholic drinks (e.g., tella and tej), eating meat and eggs, and having sexual intercourse as such practices are believed to interfere with the curative powers of the remedies.

### 3.4. Habitats and Abundance of and Threats to Medicinal Plants

Analysis of interview data revealed that majority (52.2%) of the plants used in the traditional medicinal practices of the people in the study district were harvested only from the wild, while some are harvested from home gardens and cultivated fields (Table 6). Adefa and Getaneh [38], Adefa and Abraha [14], and Amenu [40] reported similar findings in their studies conducted in the Ethiopian districts of Chencha, Tehuledere, and Chelya, respectively. Tanto et al. [55] also reported that most Ethiopian medicinal plants are harvested from the wild. According to Mander et al. [56], 56,000 tons of medicinal plants are utilized in Ethiopia annually, of which 87% is obtained from the wild. Field observations made by the present investigators revealed that there was very little effort in Raya Kobo to conserve medicinal plants. Only few medicinal plants including *Carica papaya*, *Ocimum urticifolium*, and *Rhamnus prinoides* used in treating gastric problems, mich, and infection of uvula, respectively, were purposely grown in some home gardens or cultivated fields. These indicate that the medicinal flora in the study district is under big threat due to extensive environmental degradation. According to Leta [57], deforestation and overgrazing are serious problems in Raya Kobo and other north Ethiopian places.

Of the total informants interviewed, majority (51.3%) of them attested that most of the medicinal plants were either difficult or very difficult to find, while 48.7% of them claimed that most of the medicinal plants were easy to find in the immediate environment. According to them, deforestation (49.3%), draught (22%), and overgrazing (14%) are the main factors for the depletion of medicinal plants in their places (Table 7). Most of the informants reported that they kept their knowledge of medicinal plants as secret with limited willingness to share to the younger generation. The limited interest of practitioners of traditional medicine in passing over their knowledge on medicinal plants to the younger generation is considered as another threat to the continuation of the practice in the study area. Other studies conducted elsewhere in the country also reported secrecy as a problem responsible for the loss of traditional medicinal knowledge and practices and the associated medicinal plants [20, 46, 48, 58].

Informants claimed that environmental protection/rehabilitation (60%), cultivation of medicinal plants in home gardens (14%), reforestation (10%), and demarcation of grazing lands (9.3%) are the main measures that can help in the conservation of medicinal plants in the district (Table 8). They also revealed that there were few medicinal plants that were intentionally managed in home gardens and crop fields but primarily for other purposes such as food, fodder, spice, live fence, and shade. The investigators observed that there were efforts by some traditional medical practitioners to grow/cultivate medicinal plants, which were also used as sources of food and spices, in their home gardens and crop fields. According to Asfaw [59], of the medicinal plants managed in home gardens, only 6% is cultivated for medicinal uses only. The present investigators observed that annual reforestation programs in the study district mainly focused on exotic and few indigenous plants with very limited attention to plants of medicinal values.

### 3.5. Sources of Traditional Medicinal Knowledge

Informants of this study who have participated in the interviews claimed that they acquired knowledge of medicinal plants from different sources including family members (61.3%), friends/acquaintances (14%), and traditional healers (Table 9). A study carried out in Gimbi district, western Wollega zone of Ethiopia, reported that great majority of informants (91%) cited parents as their sources of knowledge of traditional medicinal plants [60]. Other researchers have also reported similar findings elsewhere in Ethiopia [40, 61–65]. These observations imply that initiatives that promote the sharing of such knowledge have to be encouraged.

### 3.6. Acceptance of Medicinal Plant Remedies

This study revealed that treatment with medicinal plants was highly accepted in Raya Kobo because of the belief that medicinal plants were efficacious in managing different ailments. Most informants (72.7%) reported that medicinal plants are more effective as compared to modern drugs. Some informants (14.7%) claimed that there were ailments that could only be treated using medicinal plants and not with modern medications (Table 10). It is a well-known fact that traditional medicine is still recognized in different parts of the world as the preferred means to manage different ailments. Estimates show that about 80% of the Ethiopian population is still dependent on traditional medicine, which essentially involves the use of medicinal plants [4].

### 3.7. Preference Ranking of Medicinal Plants Used for Treating Mich

Analysis of preference ranking conducted on eight medicinal plants used for treating mich (febrile illness) in the study district revealed that *Ocimum urticifolium* is the most preferred plant, followed by *Withania somnifera* and *Zehneria scabra* (Table 11). Mich is an ailment in the district against which the highest number of medicinal plants was reported by the informants. Many other studies have showed that *Ocimum urticifolium* is widely used in Ethiopia for treating mich and other febrile illnesses [10, 48, 66–71]. A related species, *Ocimum lamiifolium*, is also widely employed in different parts of the country to treat mich and similar ailments [6, 10, 13, 72, 73]. The common use of *Ocimum urticifolium* and related species may imply the potency of species of the genus *Ocimum* in treating mich and febrile illnesses. Furthermore, studies conducted on medicinal properties of extracts of different *Ocimum* species showed their antipyretic properties [74–77]. *Withania somnifera* [10, 16, 26, 48, 78] and *Zehneria scabra* [8, 16, 24, 26, 79, 80] are also widely reported to be effective in treating febrile-like illnesses.

### 3.8. Comparison of Knowledge of Medicinal Plants among Different Groups

Comparison of knowledge of medicinal plants of the respondents across ages—in terms of number of remedies they reported—showed a strong correlation (*n* = 150; *r* = 0.709; *p* ≤ 0.01) (Table 12); as age increased, knowledge of medicinal plants increased. Moreover, comparisons among age groups in regard to their knowledge of medicinal plants using one-way ANOVA showed a statistically significant difference (*F* = 74.22; *p* ≤ 0.05) implying that older informants have accumulated more knowledge and experience in the study district. This finding is in agreement with results of other ethnobotanical investigations carried out elsewhere in the country [14, 62, 65, 72]. The low interest of the younger generation towards traditional medicine and medicinal plants may be influenced by the expansion of modern education and acculturation. On the other hand, results of studies by Yineger and Yewhalaw [45] and Adefa and Getaneh [38] have demonstrated the absence of correlation between the number of medicinal plant reported and the age of informants.

However, there was no significance difference in the knowledge of medicinal plants expressed in terms of mean number of prescriptions of medicinal plants reported between men and women (d*f* = 149; *F* = 0.073; *p* > 0.05). Men informants (*n* = 75) reported 617 prescriptions (mean = 8.2), while women informants (*n* = 75) reported 620 prescriptions (mean = 8.3). Similar finding was reported by Alemayehu et al. [20] in a study conducted in Minjar-Shenkora district of the Amhara State of Ethiopia. Interview results of the present study also indicated that traditional knowledge was transferred within the family without special preference to either sex.

### 3.9. Status of Knowledge of Medicinal Plants in Raya Kobo

Of the 150 informants participated in the present study, 83 (55.3%) rated themselves as having low level of knowledge of medicinal plants, while 59 (39.3%) rated themselves as having medium level of knowledge. Only eight (5.4%) informants considered themselves as having high level of knowledge of medicinal plants (Table 13). Of the 56 illiterate informants participated in the study, 5 (9%) of them reported that they have high level of knowledge of medicinal plants, and only 3 (5%) of the 62 informants with informal education claimed to have high level of knowledge. However, none of the 32 informants with formal education claimed to have high level of knowledge of medicinal plants. This may be taken as an indication that the number of people with high level of knowledge of medicinal plants in the district is dwindling due to the influence of formal education, acculturation, and expansion of modern healthcare services.

## 4. Conclusion

The result of this study showed the existence of rich traditional knowledge in Raya Kobo district on the use of medicinal plants for treating many human and livestock ailments. Since herbal preparations are cheaper and are believed to be more effective, the majority of people in the district still give priority to visit traditional healers to seek treatments for themselves and for their families and livestock. Many of the plants used for traditional remedies are collected from the wild. The observation that majority of the remedies are prepared from leaves imply that ethnomedicinal and ethnoveterinary practices in the study area do not cause the depletion of plant biodiversity. Mich, the most common ailment in the study district, is treated or managed by the highest number of medicinal plants. The preference ranking conducted on eight medicinal plants used for treating this ailment showed that *Ocimum urticifolium* is the most preferred plant. These observations are important indications of the high potency of the plant against the disease to call for prioritized scientific investigation. Generally speaking, extensive documentation of ethnomedicinal and ethnoveterinary knowledge has to be pursued in the study district and neighboring regions before it is too late.

## Figures and Tables

**Table 1:** Medicinal plants used to treat human ailments.

Scientific name	Local name	Voucher number	Habit	Ailment treated	Plant part used	Preparation methods	Administration route	No. of informants
*Acacia abyssinica* Hochst. ex Benth. (Fabaceae)	Grar	AS-73-2017	Tree	Allergy	Leaf	Pounding and then squeezing	Dermal	11
				Broken bone	Root	Crushing	Dermal on the broken bone	15
*Acokanthera schimperi* (A.DC.) Schweinf. (Apocynaceae)	Merez	AS-69-2017	Tree	Itch	Stem bark	Drying and then grinding	Dermal	12
*Allium cepa* L. (Alliaceae)	Key shinkurt	AS-78-2017	Herb	Stomach complaints	Bulb (leaf)	Pounding after mixing it with *A. sativum* and *Ruta chalepensis*, and then adding honey	Oral	7
				Cough				8
*Allium sativum* L. (Alliaceae)	Nech shinkurt	–	Herb	Stomach complaints	Bulb (leaf)	Pounding or chewing	Oral	10
				Headache	Bulb (leaf)	Pounding and chewing	Oral	11
				Malaria	Bulb (leaf)	Pounding after mixing it with *Cicer arietinum*	Oral	5
				Cough	Bulb (leaf)	Pounding or crushing and then mixing it with honey	Oral	14
				Amoeba	Bulb (leaf)	Grinding and then mixing it with honey	Oral	6
				Lung cancer/ tuberculosis	Bulb (leaf)	Grinding and then mixing it with honey	Oral	4
*Aloe* spp.(Aloaceae)	Eret	AS-29-2017	Herb	Wound	Stem	Cutting to harvest the jelly juice	Dermal	16
				Malaria	Stem	Cutting to harvest the jelly juice	Oral	12
				Diarrhea	Root	Cutting to harvest the jelly juice	Oral	13
*Alternanthera pungens* Kunth (Amaranthaceae)	Yemeret Kitigne/get	AS-58-2017	Herb	Wound	Leaf	Grinding or pounding	Dermal on the wounded part	6
*Argemone mexicana* L. (Papaveraceae)	Enkushashle	AS-57-2017	Herb	Sore	Stem	Cutting to harvest the latex	Dermal on the affected part	11
*Artemisia absinthium* L. (Asteraceae)	Natra	AS-22-2017	Herb	Uvula infection	Whole plant	Squeezing and producing juice	Oral	4
*Azadirachta indica* A. Juss. (Meliaceae)	Nim	AS-41-2017	Tree	Cough	Leaf	Boiling in water after mixing it with *Eucalyptus globulus*	Inhalation (oral and nasal)	10
*Balanites aegyptiaca* (L.) Delile (Zygophyllaceae)	Bedena	AS-61-2017	Tree	*Tinea nigra*	Leaf	Crushing	Dermal on the affected part	5
				Stomach complaints	Fruit	Chewing	Oral	7
				Bloody diarrhea	Leaf	Crushing to collect juice	Oral	8
*Brassica nigra* L. (Brassicaceae)	Senafich	—	Herb	Stomach complaints	Seed	Dying, then grinding after mixing it with *A. sativum* and *Vicia faba*	Oral	6
*Biancaea decapetala* (Roth) O. Deg. (Fabaceae)	Kentefa	AS-70-2017	Shrub	Evil eye	Leaf and root	Pounding it after mixing it with *Ruta chalepensis*	Oral	8
*Calotropis procera* (Aiton) W.T. Aiton (Apocynaceae)	Topia	AS-48-2017	Shrub	Wart	Leaf	Cutting to harvest the latex	Dermal on the affected part	9
*Carica papaya* L. (Caricaceae)	*Papaye*	AS-55-2017	Herb	Swelling on the skin	Fruit, seeds	Splitting	Dermal on the swollen part	8
*Carissa spinarum* L. (Apocynaceae)	Agam	AS-30-2017	Shrub	Mental stress	Root	Boiling it in water	Dermal	3
				Devil's illness	Leaf	Boiling it in water after mixing with *Croton macrostachyus* roots	Inhalation (oral and nasal)	7
				Snake bite	Leaf	Pounding after mixing with leaves of *Verbena officinalis*	Oral	5
*Caylusea abyssinica* (Fresen.) Fisch. & C.A. Mey. (Resedaceae)	Kibezelzil	AS-64-2017	Herb	Mich	Leaf	Boiling it in water	Inhalation (oral and nasal)	15
*Chenopodiastrum murale* (L.) S. Fuentes, Uotila & Borsch (Amaranthaceae)	Amedmado	AS-75-2017	Herb	Broken bone	Leaf	Crushing	Dermal on the broken bone	5
*Citrullus lanatus* (Thunb.) Matsum. & Nakai (Cucurbitaceae)	Habhab	—	Herb	Hypertension	Fruit	Squeezing	Oral	4
				Stomach complaints	Fruit	Slicing	Oral	9
*Citrus limon* (L.) Osbeck (Rutaceae)	Lomi	AS-59-2017	Tree	Stomach ache	Fruit	Squeezing it to produce juice and then adding honey	Oral	16
*Clematis hirsuta* Perr. & Guill. (Ranunculaceae)	Chicho	AS-67-2017	Herb	Cancer	Leaf	Crushing	Dermal on the affected part	7
*Clematis simensis* Fresen. (Ranunculaceae)	Azo hareg	AS-36-2017	Herb	Cancer	Leaf	Crushing	Dermal on the affected part	4
*Coffea arabica* L. (Rubiaceae)	Buna	AS-46-2017	Shrub	Wound	Seed	Roasting and pounding	Dermal on wound	12
				Asthma	Seed	Roasting and pounding	Oral	6
*Cordia africana* Lam. (Boraginaceae)	Wanza	AS-62-2017	Tree	Hepatitis/jaundice	Leaf	Boiled in water after mixing it with *Sorghum bicolor*, chewing	Oral	8
				*Mich*	Leaf	Boiling it in water	Inhale (oral and nasal)	10
*Croton macrostachyus* Hochst. ex Delile (Euphorbiaceae)	Mekenisa	AS-19-2017	Tree	*Tinea nigra*	Leaf	Grinding or pounding	Dermal	4
				*Mich*	Leaf	Boiling it in water	Inhale (oral and nasal)	7
*Cucumis dipsaceus* C.G. Ehrenb. ex Spach (Cucurbitaceae)	Yemdir embuay	AS-13-2017	Herb	Wound	Fruit	Crushing	Dermal	10
				Jaundice/hepatitis	Whole plant	Chewing or crushing after adding water and then filtering	Dermal	4
*Cynoglossum lanceolatum* Forssk. (Boraginaceae)	Yegid Zemedie	AS-17-2017	Herb	Mich	Leaf	Squeezing	Auricular	12
				Abdominal pain	Leaf	Chewing	Oral	4
*Datura stramonium* L. (Solanaceae)	Banjie	AS-24-2017	Herb	Hemorrhoids	Leaf	Squeezing	Dermal	6
*Dyschoriste radicans* Nees (Acanthaceae)	Telba Titi	AS-21-2017	Herb	Anthrax	Whole plant	Crushing	Dermal on the swollen part	3
*Eucalyptus globulus* Labill. (Myrtaceae)	Nech bahirzaf	AS-72-2017	Tree	Common cold	Leaf	Boiling in water after mixing it with *Withania somnifera*	Inhalation (oral and nasal)	14
				Devil's illness				8
*Euclea divinorum* Hiern. (Ebenaceae)	Dedoho	AS-26-2017	Shrub	Mental stress	Root	Boiling in water	Oral	3
				Joint ache	Stem	Putting it on fire to yield smoke	Fumigation (nasal)	8
				Evil eye	Whole	Crushing	Dermal around the neck	7
				Snake bite	Root bark	Crushing and pounding after adding water	Oral	3
				Black spider bite	Root	Chewing	Oral	9
*Euphorbia* sp. (Euphorbiaceae)	Qulqualda	AS-38-2017	Shrub	Thorn inside the skin	Leaf	Cutting to harvest the latex	Dermal on the affected skin	10
				Cancer	Leaf	Cutting to harvest the latex	Dermal (tying it on the wound)	3
*Euphorbia tirucalli* L. (Euphorbiaceae)	Kinchibt	AS-53-2017	Shrub	Hemorrhoids	Stem	Cutting to harvest the latex	Dermal on the affected part	3
				Wart	Stem	Cutting to harvest the latex	Dermal on the infected part	8
*Ficus palmata* Forsskål (Moraceae)	Beles	AS-16-2017	Shrub	Wound	Leaf	Grinding or pounding	Dermal	9
				*Tinea nigra*	Leaf	Cutting the leaves and harvesting the latex	Dermal	6
*Guizotia abyssinica* (L.f.) Cass. (Asteraceae)	Nug	—	Herb	Gastritis	Seed	Pounding	Oral	12
*Hagenia abyssinica* Willd. (Rosaceae)	Kosso	—	Tree	Stomach distention	Root	Grinding and stirring the powder in tella (locally produced drink)	Oral	16
*Heliotropium cinerascens* A. DC. (Boraginaceae)	Nechilo	AS-39-2017	Shrub	*Mich*	Leaf	Boiling it in water	Inhale (oral and nasal)	15
				Wound	Leaf	Boiling it in water after mixing it with *Zehneria scabra*	Dermal on the affected part	7
*Hydnora johannis* Becc. (Hydnoraceae)	Demerech	AS-77-2017	Herb	Wound	Root	Crushing	Dermal on the wounded part	8
*Jasminum grandiflorum* L. (Oleaceae)	Tembelel	AS-14-2017	Shrub	Snake bite	Leaf	Crushing and squeezing to collect juice	Oral	6
				Anthrax	Leaf	Drying and then pounding	Dermal	3
*Juniperus procera* Hochst. ex Endl. (Cupressaceae)	Yehabesha-tsid	—	Herb	Cough	Stem/root	Crushing or grinding and putting it on fire or boiling it in water	Fumigation or inhalation	7
*Justicia schimperiana* (Hochst. ex Nees) T. Anders (Acanthaceae)	Simiza	AS-34-2017	Shrub	Mental stress	Root	Boiling it in water	Dermal	3
				Mich	Leaf	Chewing	Oral	11
				Jaundice	Leaf	Squeezing	Oral	6
*Lawsonia inermis* L. (Lythraceae)	Hina	AS-54-2017	Shrub	Rheumatic disease	Leaf	Pounding it after mixing it with *Citrus limon* and adding water	Oral	3
*Lepidium sativum* L. (Brassicaceae)	Feto	—	Herb	Stomach complaints	Seed	Grinding and mixing it in water	Oral	15
*Leucas abyssinica* (Benth.) Briq. (Lamiaceae)	Aftegegne	AS-03-2017	Shrub	*Mich*	Leaf	Crushing	Oral	10
*Malva parviflora* L. (Malvaceae)	Zebenya	AS-12-2017	Herb	Diarrhea	Leaf	Pounding	Oral	9
				*Mich*	Leaf	Squeezing	Oral	14
*Mangifera indica* L. (Anacardiaceae)	Mango	AS-56-2017	Tree	*Mich*	Leaf	Roasting	Dermal	12
*Mentha* × *piperita* L. (Lamiaceae)	Nana	AS-40-2017	Herb	Diarrhea	Leaf, stem	Pounding after mixing it with *Nigella sativa* and *A. sativum*	Oral	8
*Moringa stenopetala* (Baker f) Cufod. (Moringaceae)	Sheferaw	AS-43-2017	Tree	Hypertension	Leaf	Drying and Pounding, and filtering it after adding water	Oral	6
*Musa* × *paradisiaca* L. (Musaceae)	Muz	—	Shrub	Rough skin	Fruit	Peeling to remove the skin	Dermal	10
*Myrsine africana* L. (Primulaceae)	Kechem	AS-23-2017	Shrub	Tapeworm	Fruit	Crushing	Oral	4
*Nigella sativa* L. (Ranunculaceae)	Tikur Azmud	—	Herb	Stomach complaints	Seed	Pounding it after mixing it with *A. sativum*, *Ruta chalepensis*, and *A. cepa* and then adding lemon juice	Oral	12
*Ocimum urticifolium* Roth (Lamiaceae)	Dema Kassie	AS-42-2017	Shrub	*Mich*	Leaf	Boiling in water after mixing it with *Withania somnifera*	Oral	15
				Common cold	Leaf		Inhale (oral and nasal)	8
				Hypertension	Leaf		Inhale (oral and nasal)	6
*Olea europaea* L. subsp. *cuspidata* (Wall. & G. Don) Cif. (Oleaceae)	Weyra	AS-49-2017	Tree	Uvula infection	Leaf	Pounding and then chewing	Oral	9
				Wound	Leaf	Squeezing	Dermal on the infected part	7
*Oxalis radicosa* A. Rich. (Oxalidaceae)	Shimburut	AS-07-2017	Herb	Snake bite	Root	Crushing	Oral	8
*Phytolacca dodecandra* L'Hér. (Phytolaccaceae)	Mehan endod	AS-25-2017	Shrub	Snake bite	Root	Chewing	Oral	4
*Plectranthus* spp. (Lamiaceae)	Tezeteza	AS-20-2017	Herb	Bleeding	Leaf	Crushing	Nasal (smelling)	8
*Rhamnus prinoides* L'Hér. (Rhamnaceae)	Gesho	AS-33-2017	Shrub	Tonsillitis	Leaf	Crushing or chewing	Oral	14
				Uvula infection	Leaf	Squeezing to produce juice	Oral	7
*Ricinus communis* L. (Euphorbiaceae)	Gulo	AS-47-2017	Shrub	Devil's illness	Leaf	Pounding after mixing it with *Withania somnifera* and squeezing	Oral	10
*Rumex abyssinicus* Jacq. (Polygonaceae)	Moqmoqo	—	Shrub	Common cold	Root	Burning	Fumigation (oral and nasal)	15
*Rumex nervosus* Vahl (Polygonaceae)	Embacho	AS-31-2017	Shrub	Mental stress	Root	Boiling it in water	Dermal	7
*Ruta chalepensis* L. (Rutaceae)	Tena Adam	AS-32-2017	Herb	Asthma	Leaf	Adding it into a boiled tea/coffee	Oral	12
				Stroke	Leaf/seed	Pounding or crushing	Oral	3
				Stomach ache	Leaf/stem	Crushing	Oral	11
				Devil's illness	Leaf	Unprocessed	Nasal (smelling)	16
*Schinus molle* L. (Anacardiaceae)	Qundo berberie	AS-60-2017	Tree	Mich	Leaf	Boiling it in water after mixing it with *Psychotria* sp.	Inhalation (oral and nasal)	15
*Sida schimperiana* Hochst. ex A. Rich. (Malvaceae)	Chifrig	AS-05-2017	Shrub	Toothache	Leaf	Grinding	Oral	7
				Erectile dysfunction	Root	Mixing it with *Nigella sativa*, *A. sativum,* and honey and boiling it in water	Oral	8
				Evil eye	Root	Chewing	Oral	9
*Silene macrosolen* A. Rich. (Caryophyllaceae)	Wegert	—	Shrub	Devil's illness	Root	Putting it on fire to produce smoke	Fumigation (oral and nasal)	8
*Solanum marginatum* L.f. (Solanaceae)	Embuay	AS-02-2017	Shrub	Stomach illness	Root	Pounding by mixing it with root of *Tragia* sp.	Oral	6
				Snake bite	Root	Crushing	Oral	8
*Terminalia brownii* Fresen. (Combretaceae)	Inkoy	AS-65-2017	Shrub	Uvula infection	Leaf	Squeezing	Oral	9
*Tragia* spp. (Euphorbiaceae)	Awl alit	AS-11-2017	Herb	Wound	Leaf	Crushing	Dermal	9
				Evil eye	Root	Grinding or pounding	Dermal	6
*Trigonella foenum-graecum* L. (Fabaceae)	Abish	—	Herb	Evil eye	Leaf	Crushing and adding it on fire	Fumigation (nasal)	10
				Broken leg	Seed	Grinding or pounding or boiling it in water	Dermal on the broken bone	6
*Verbascum sinaiticum* Benth. (Scrophulariaceae)	Yejib Chama	AS-06-2017	Herb	Mental stress	Root	Squeezing and boiling it in water	Dermal	5
				Uterus retention	Root	Squeezing and boiling it in water	Nasal	7
*Vicia faba* L. (Fabaceae)	Bakela	—	Herb	Swelling on skin	Seed	Grinding or pounding	Dermal	15
				Cough		Chewing	Oral	10
*Vitis vinifera* Linn. (Vitaceae)	Wein	—	Tree	Eye diseases (dirt)	Seed	Pounding and then squeezing	Ocular	5
*Withania somnifera* (L.) Dunal (Solanaceae)	Gizewa	AS-35-2017	Shrub	*Mich*	Root	Drying and pounding	Fumigation (nasal)	12
					Root	Chewing	Oral	8
				Evil disease	Leaf	Boiling it in water	Inhalation (nasal)	16
*Zehneria scabra* (L.f.) Sond. (Cucurbitaceae)	Hareg resa	AS-45-2017	Herb	*Mich*	Leaf	Boiling it in water	Inhalation (oral and nasal)	12
*Zingiber officinale* Roscoe (Zingiberaceae)	Zingible	—	Herb	Tonsillitis	Rhizome (stem)	Chewing	Oral	6
				Common cold		Pounding and boiling	Inhale (oral, nasal)	15
*Ziziphus spina-christi* (Rhamnaceae)	Qunqura	AS-52-2017	Tree	Devil's illness	Leaf	Pounding and then squeezing	Oral	7
				Dandruff	Leaf	Pounding after adding water and then squeezing	Dermal on the affected part	13

**Table 2:** Medicinal plants used to treat livestock ailments.

Scientific name	Local name	Voucher number	Habit	Ailment treated	Plant part used	Preparation method	Administration route	No. of informants
*Acacia* spp. (Fabaceae)	Doret	AS-28-2017	Shrub	Wound	Leaf	Crushing	Dermal	7
				Eye infection	Leaf	Chewing	Ocular	8
*Bidens prestinaria* (Sch. Bip.) Cufod. (Asteraceae)	Chigogot	AS-18-2017	Herb	Insect bite	Leaf	Pounding to produce juice	Dermal	6
*Calpurnia aurea* (Aiton) Benth. (Fabaceae)	Digita	AS-04-2017	Herb	Mange	Leaf	Crushing	Dermal	10
*Chenopodium ambrosioides* L. (Chenopodiaceae)	Sinign	AS-09-2017	Herb	Chicken flea	Root	Putting it on fire	Fumigating the house	4
*Cissus quadrangularis* L. (Vitaceae)	Kimtita	AS-44-2017	Herb	Mange	Whole plant	Crushing	Dermal	11
*Cyphostemma adenocaule* (Steud. ex A. Rich.) Desc. ex Wild & R.B. Drumm. (Vitaceae)	Aba Woldu	AS-10-2017	Herb	Leech infestation	Root	Crushing	Oral	13
*Dodonaea angustifolia* L.f. (Sapindaceae)	Kitkita	AS-50-2017	Shrub	Bone dislocation	Leaf	Crushing and pounding	Dermal	4
*Echinops kebericho* Mesfin (Asteraceae)	Kebercho	—	Herb	Anthrax	Root	Grinding to yield juice	Oral	10
*Echinops* spp. (Asteraceae)	Kushelie	AS-27-2017	Shrub	Bone dislocation	Root	Unprocessed	Dermal (tie it on the dislocated bone)	14
*Ficus vasta* Forssk. (Moraceae)	Warka	AS-74-2017	Tree	Thrips	Stem bark	Pounding and boiling it in water	Oral	7
*Kalanchoe marmorata* Bak. (Crassulaceae)	Endahul	AS-01-2017	Herb	Cancer	Root	Crushing	Dermal	4
*Lycopersicon esculentum* Mill. (Solanaceae)	Timatim	—	Herb	Leech infestation	Leaf	Pounding after adding water	Oral and nasal	14
*Maytenus senegalensis* (Lam.) Exell (Celastraceae)	Qoqoba	AS-71-2017	Shrub	Insect bite	Leaf	Crushing it to yield juice	Dermal	10
*Nicotiana tabacum* L. (Solanaceae)	Timbaho	AS-15-2017	Shrub	Leech infestation	Leaf	Squeezing or pounding after adding water	Oral and nasal	8
*Otostegia integrifolia* Benth. (Lamiaceae)	Tunjiut	AS-68-2017	Shrub	Wound	Leaf	Crushing	Dermal on the wound	10
				Ear disease	Leaf	Pounding and squeezing to yield juice	Auricular	7
				Flea infestation	Leaf	Putting it on fire	Fumigating house	7
*Portulaca oleracea* L. (Portulacaceae)	Antaria	AS-63-2017	Herb	Bone dislocation	Root	Crushing	Dermal on the dislocated bone	10
*Ximenia americana* L. (Olacaceae)	Ikma	AS-66-2017	Shrub	Emaciation	Stem bark	Boiling it in water	Oral	6

**Table 3:** Human diseases and the corresponding number of medicinal plants used for their treatments.

SN	Ailment treated	No. of medicinal plants used
1	Gastrointestinal complaints	17
2	*Mich* (febrile illness)	13
3	Wound	10
4	Snake bite	6
5	Devil's illness	6
6	Mental stress	5
7	Dislocated/broken bone	5
8	Cough	5
9	Evil eye	5
10	Common cold	4
11	Uvula infection	4
12	Tinea nigra	3
13	Jaundice	3
14	Cancer	3
15	Hemorrhoids	2
16	Swelling on the skin	2
17	Malaria	2
18	Tonsillitis	2
19	Wart	2
20	Asthma	2
21	Allergy	1
22	Thorn inside the skin	1
23	Toothache	1
24	Hypertension	1
25	Anthrax	1
26	Hepatitis	1
27	Headache	1
28	Itch	1
29	Bleeding	1
30	Dandruff	1
31	Erectile dysfunction	1
32	Joint ache	1
33	Black spider bite	1
34	Eye disease	1
35	Lung disease	1
36	Rough skin	1
37	Stroke	1
38	Uterus retention	1

**Table 4:** Livestock diseases and the corresponding number of medicinal plants used for their treatments.

SN	Ailment treated	No. of medicinal plants used
1	Dislocated/broken bone	3
2	Leech infestation	3
3	Mange	2
4	Insects bites	2
5	Wound	2
6	Anthrax	1
7	Cancer	1
8	Eye infection	1
9	Thrips	1
10	Flea infestation	1
11	Ear disease	1
12	Emaciation	1

**Table 5:** Remedy preparation methods in Raya Kobo district.

SN	Remedy preparation method	Number of preparations	Percentage
1	Grinding/pounding	31	23.7
2	Crushing	26	19.8
3	Boiling	19	14.5
4	Chewing	14	10.7
5	Squeezing	11	8.4
6	Cutting and bleeding	9	6.9
7	Pounding and squeezing	4	3.1
8	Burning	3	2.3
9	Squeezing and boiling	2	1.5
10	Roasting and pounding	2	1.5
11	Pounding and chewing	2	1.5
12	Splitting and slicing	2	1.5
13	Crushing and squeezing	1	0.8
14	Roasting	1	0.8
15	Crushing and burning	1	0.8
16	Pounding and boiling	1	0.8
17	Peeling	1	0.8
18	Unprocessed	1	0.8
	Total	131	100

**Table 6:** Habitats of medicinal plants in Raya Kobo district.

SN	Habit of the plants	Number of medicinal plants	Percentage
1	Wild only	48	52.70
2	Home garden only	18	19.80
3	Cultivation field only	16	17.60
4	Wild or home garden	6	6.60
5	Wild or cultivation land	3	3.30
	Total	91	100.0

**Table 7:** Reasons for depletion of medicinal plants in Raya Kobo district as reported by informants.

SN	Reasons for depletion of medicinal plans	Number of informants	Percentage
1	Deforestation	74	49.3
2	Drought	33	22.0
3	Over‐grazing	21	14.0
4	Overexploitation	8	5.3
5	Firewood collection	8	5.3
6	Environmental degradation	3	2.0
7	Other factors	3	2.0
	Total	150	100.0

**Table 8:** Ways of conserving medicinal plants in Raya Kobo district.

SN	Conservation activity	Percentage
1	Environmental protection/rehabilitation	60.0
2	Cultivation in home gardens	14.0
3	Reforestation	10.0
4	Isolation of grazing lands	9.3
5	Cultivation on agricultural plots	5.3
6	Construction of check dams	1.3
	Total	100.0

**Table 9:** Sources of traditional knowledge on medicinal plants in Raya Kobo district.

SN	Sources of knowledge	Frequency	Percentage
1	Family members	92	61.3
2	Friends/acquaintances	21	14.0
3	Traditional healers	17	11.3
4	Books	7	4.7
5	Observation	6	4.0
6	Relatives	5	3.3
7	Modern health practitioners	1	0.7
8	Others	1	0.7
	Total	150	100.0

**Table 10:** Reasons reported by informants in the district for acceptance of traditional medicinal plants.

SN	Reasons for acceptance of medicinal plants	Frequency	Percentage
1	Effectiveness	109	72.7
2	Ailment not treated with modern medication	22	14.7
3	Easy accessibility	8	5.3
4	Cheaper cost	7	4.7
5	Better efficacy than modern medication	2	1.3
6	Absence of modern healthcare facility nearby	1	0.7
7	Other reasons	1	0.7
	Total	150	100

**Table 11:** Preference ranking exercise conducted on eight plants used to treat mich in Raya Kobo district.

SN	Species	Respondents							Total	Rank
		1	2	3	4	5	6	7		
1	*Azadirachta indica*	7	6	6	7	6	7	6	45	5
2	*Cynoglossum lanceolatum*	5	6	4	6	7	4	6	38	8
3	*Eucalyptus globulus*	6	7	8	7	7	5	7	47	4
4	*Heliotropium cinerascens*	8	7	6	7	6	5	6	43	6
5	*Justicia schimperiana*	6	7	5	7	7	6	7	41	7
6	*Ocimum urticifolium*	8	7	8	8	8	7	8	54	1
7	*Withania somnifera*	8	7	7	8	6	7	8	51	2
8	*Zehneria scabra*	8	8	7	7	6	6	8	50	3

**Table 12:** Correlations of informants' ages and number of medicinal plant citation.

		No. of plant citations	Age of informants
No. of plant citations	Pearson correlation	1	0.709^*∗∗*^
	Significance (2-tailed)		0.000
	*N*	150	150
Age of informants	Pearson correlation	0.709^*∗∗*^	1
	Significance (2-tailed)	0.000	
	*N*	150	150

^*∗∗*^Correlation is significant at the 0.01 level (2-tailed).

**Table 13:** Levels of education and medicinal plants knowledge reported by informants in Raya Kobo.

SN	Educational level	Knowledge of traditional medicinal plants			Total
		Low	Medium	High	
1	Illiterate	31 (20.7%)	20 (13.3%)	5 (3.3%)	56 (37.3%)
2	Informal education	36 (24.0%)	23 (15.3%)	3 (2.0%)	62 (41.3%)
3	Formal education	16 (10.7%)	16 (10.7%)	0 (0.0%)	32 (21.3%)

	Total	83 (55.3%)	59 (39.3%)	8 (5.3%)	150 (100)

## Data Availability

The data used to support this study are available from the corresponding author upon request.
